# Response to letter regarding ‘The impacts of partial replacement of red and processed meat with legumes or cereals on protein and amino acid intakes: a modelling study in the Finnish adult population’

**DOI:** 10.1080/07853890.2024.2361816

**Published:** 2024-06-04

**Authors:** Meri Simojoki, Satu Männistö, Heli Tapanainen, Mirkka Maukonen, Liisa M. Valsta, Suvi T. Itkonen, Anne-Maria Pajari, Niina E. Kaartinen

**Affiliations:** a Department of Public Health and Welfare, Finnish Institute for Health and Welfare (THL), Helsinki, Finland; b Department of Food and Nutrition, University of Helsinki, Helsinki, Finland

Dear Editor,

We thank Mariotti et al. for the valuable comments in their letter to the editor regarding our published article ‘The impacts of partial replacement of red and processed meat with legumes or cereals on protein and amino acid intakes: a modelling study in the Finnish adult population’ [1]. The letter focused on the proportions below estimated average requirement (EAR) for protein and amino acid intakes as well as on methodological issues related to energy underreporting.

Mariotti et al. compare our results to their modelling study in France [2] and point out that the proportions below the EAR for protein and amino acid intakes are much higher in our study compared to their approach. In the reference diet of our study, the proportions below the EAR for protein intake were 5–7% in participants aged 18–64 years. In the French study, the corresponding proportion was 0.3%.

Possible reasons for the differences in protein adequacy include differences in the characteristics of the study population, food consumption of the study population, dietary assessment method, or study design. In our study, we used data from the National FinDiet 2017 Survey of 1655 Finnish adults (780 men and 875 women) aged 18–74 years [3]. The dietary data were collected using two non-consecutive 24-h recalls. In the French study, the dietary data were derived from the 7-day food records of 1678 French adults (717 men and 961 women) aged 18–65 years. Both studies used usual intake modelling methodology to estimate protein and amino acid intakes against EAR. The EAR values were those estimated by the FAO/WHO/UNU.

As Mariotti et al. pointed out, energy underreporting is a potential source of error in self-reported dietary assessment methods. We included underreporters and overreporters in our study, while the French study excluded them. In the FinDiet 2017 Survey, the rate of energy underreporting was 21% (23% for men and 20% for women) in the in-person interview and 18% (21% for men and 15% for women) in the telephone interview. Energy overreporting was below 0.5%. The rate of energy underreporting was at the same level or lower than in 24-h dietary recalls in general [4].

Our results describe the observed and modelled intake of protein and amino acids based on two non-consecutive 24-h recalls. All interviewed days were included irrespective of the energy intake, which naturally varies in this type of surveys. We focused on changes in protein and amino acid intakes when partially replacing red and processed meat with legumes or cereals in the diet and remained cautious in our conclusions regarding the percentages below the EAR.

Mariotti et al., in their letter, estimated the potential impact of energy underreporting in our study. We also made new calculations to assess the impact. We calculated proportions below the EAR for protein intake in the reference diet without underreporters. When underreporters were excluded, the proportions below the EAR for protein intake decreased to <1% in both men and women aged 18–64 years. Therefore, energy underreporting may explain most of the differences between studies, as estimated by Mariotti et al.

Unlike the French study, however, our study also assessed the adequacy of protein intake in elderly population. When underreporters were excluded, 4% of both men and women aged 65–74 years still had protein intake below the EAR. Therefore, protein intake in elderly population needs attention.

In addition, besides energy underreporting, the body weight of participants may partly explain the differences between studies. The EAR for protein intake estimated by FAO/WHO/UNU (0.66 g/kg body weight) takes body weight into account similarly in both studies. The proportion of overweight or obese adults (body mass index ≥25 kg/m^2^) is 63% in Finland, compared to 47% in France [5,6]. In an overweight or obese study population, the proportion below the EAR for protein intake is likely to be higher.

As Mariotti et al. remarked, the lysine content of the cereal aggregate impacted the lysine intake in our study. The most consumed cereal food in Finland is rye bread, which accounted for 59% of the cereal aggregate used in our study. Rye bread is rich in lysine compared to other cereal foods, which may partly explain the difference between studies.

We studied the impacts of partial replacement of red and processed meat with legumes or cereals on protein and amino acid intakes using a feasible approach. Replacements were made in grams per gram to simulate a real-life change in diet. Although our study did not use isoenergetic replacement scenarios, there was no meaningful difference in energy intake between the reference diet and the scenarios.

Overall, we observed that changes in protein and amino acid intakes against EAR were relatively small when red and processed meat was partly replaced with legumes or cereals. Previous modelling study using the same dataset and study design investigated the adequacy of other nutrients [7]. We agree with Mariotti et al. that the nutrients of concern in plant-based diets of working-age population are neither protein nor amino acids, while we also notice the risk of protein inadequacy in the elderly. Our results can be used for designing adequate nutrition for all population groups in transition towards a more plant-based and sustainable diet.

## Data Availability

The data of the Finnish Institute for Health and Welfare (THL) are openly available to other researchers for research purposes on a contractual basis.
